# A Novel Deep Learning Framework for Liver Fibrosis Staging and Etiology Diagnosis Using Integrated Liver–Spleen Elastography

**DOI:** 10.3390/diagnostics15232986

**Published:** 2025-11-24

**Authors:** Kai Yang, Fei Chen, Aiping Tian, Long Deng, Xiaorong Mao

**Affiliations:** 1 The First Clinical Medical College of Lanzhou University, Lanzhou 730000, China; ykai011027@163.com (K.Y.); lzhchf@163.com (F.C.); ldyy_longdeng@lzu.edu.cn (L.D.); 2 Department of Ultrasound, The First Affiliated Hospital of Lanzhou University, Lanzhou 730000, China; 3 Department of Infection, The First Affiliated Hospital of Lanzhou University, Lanzhou 730000, China; tapsea@163.com

**Keywords:** liver fibrosis, transfer learning, machine learning, two-dimensional shear wave elastography

## Abstract

**Objectives:** Liver fibrosis staging and etiology diagnosis are critical for patient management, but non-invasive methods remain challenging. This study aims to evaluate the performance of radiomics models using 2D shear wave elastography (2D-SWE) of the liver and spleen for liver fibrosis staging and etiology differentiation, comparing them with serum biomarkers and conventional ultrasound. **Methods:** A retrospective analysis was conducted on 198 patients with liver fibrosis confirmed by biopsy. Radiomics features were extracted from the liver and spleen grayscale and 2D-SWE images. Machine learning (ML) and transfer learning (TL) models were established for fibrosis staging and etiology diagnosis. Model performance was evaluated according to receiver operating characteristic (ROC) curves. **Results:** For fibrosis staging, 2D-SWE-based models outperformed grayscale and serum biomarkers. The combined liver–spleen TL model achieved exceptional validation performance (AUCs 0.99 for S4, 0.98 for ≥S3, 1.00 for ≥S2). For etiology diagnosis, the liver 2D-SWE TL model and the combined liver–spleen TL model achieved AUCs of 0.97 and 0.94, respectively, significantly outperforming ML models in terms of AUC. **Conclusions:** Integrating liver and spleen 2D-SWE radiomics with TL significantly improves non-invasive liver fibrosis staging and etiology diagnosis, offering superior accuracy over conventional methods. This approach holds promise for clinical application, though further validation is needed.

## 1. Introduction

Liver fibrosis is a common outcome of various chronic liver diseases, and its etiologies vary by region. In Western countries, the primary causes are alcoholic liver disease and metabolic dysfunction-associated steatotic liver disease, while in the Asia-Pacific region, chronic viral hepatitis is predominant [1]. As the final stage of liver fibrosis, liver cirrhosis has become the 11th leading cause of death worldwide, imposing a heavy healthcare burden. Therefore, early and accurate staging of liver fibrosis serves as a crucial basis for treatment and prognostic management [2].

Liver biopsy is the gold standard for liver fibrosis staging, but due to its invasive nature, it carries a risk of complications and is limited by sampling errors and inter-observer variability [3]. This limitation has driven the development of non-invasive detection methods for liver fibrosis, including serum biomarkers and imaging techniques. Among these, serological indicators such as aspartate transaminase-to-platelet ratio index (APRI) and fibrosis index based on four factors (FIB-4) are widely used in the diagnosis of severe liver fibrosis or liver cirrhosis, but they have difficulty distinguishing between different fibrosis stages and are easily affected by other systemic diseases [4]. With the advancement of ultrasound technology, liver stiffness measurement based on elastography has become an important assessment method, and it has attracted significant attention due to its high efficiency and repeatability [2,5].

2D-shear wave elastography (2D-SWE) employs acoustic radiation force pulses to perform continuous multi-point focusing in tissues, generating an approximately cylindrical shear wave cone. Its efficiency is 4 to 8 times that of conventional wave sources, and it effectively avoids the limitations of transient elastography caused by factors such as ascites, high body mass index, and narrowed intercostal spaces [6,7]. This technology has been widely applied in the non-invasive diagnosis of liver fibrosis, achieving promising results [8,9]. It is worth noting that spleen stiffness reflects the increased portal vein pressure caused by liver fibrosis, which in turn leads to splanchnic circulation congestion and structural changes in the spleen. This makes spleen stiffness a direct surrogate marker for portal hypertension and, at the same time, an indirect indicator of the severity of liver fibrosis [10,11,12]. However, this technology is still constrained by several factors, such as the selection of the optimal region of interest, the operator’s experience, obesity, transaminase levels, as well as steatosis and necroinflammatory activity, leading to instability in its diagnostic performance [13,14,15].

With the emergence and popularization of radiomics technology, the field of medical image analysis has grown exponentially. It converts images into mineable data by extracting a large number of image features, enabling the discovery of subtle disease characteristics that are overlooked by visual observation [16,17]. Numerous studies have confirmed the feasibility and potential of using radiomics for quantitative analysis of ultrasound images [18,19,20]. In recent years, the integration of deep learning has further expanded the field of radiomics, which includes studies based on convolutional neural network (CNN) models and those adopting transfer learning (TL). Studies have confirmed that the performance of liver fibrosis staging diagnosis based on CNN models is significantly superior to that of other conventional non-invasive methods, and it holds high clinical application value [21,22]. In addition, Liu et al. [23] established a deep learning model by combining multiple non-invasive techniques, whose efficacy is higher than that of a single technique. TL improves diagnostic performance by transferring knowledge from other fields to the medical field. Xue et al. [24] established a model based on the Inception-V3 network, and their study confirmed that the model constructed using TL had higher accuracy in diagnosing liver fibrosis compared to those without TL. Yu et al. [25] investigated the rats’ fibrosis scoring by transfer learning with AlexNet and compared them against conventional non-deep learning-based algorithms. Furthermore, the effectiveness of transfer learning technology has also been confirmed in different organs [26,27,28].

Unlike the aforementioned studies, the core contribution of this study lies in proposing a TL framework that integrates 2D-SWE of the liver and spleen. By integrating multi-organ imaging, this framework enables integrated diagnosis of liver fibrosis staging and etiological identification. Through systematic comparative validation of the proposed model against various traditional machine learning methods and serum biomarkers, this study provides a reliable performance benchmark for the field of liver disease radiomics. Finally, with its non-invasive and high-precision diagnostic efficacy, this framework demonstrates significant potential for clinical translation, offering robust methodological support for reducing reliance on liver biopsy and advancing individualized liver disease management.

## 2. Methods

### 2.1. Patients

A retrospective analysis was performed on data from 198 liver fibrosis patients admitted to our hospital between May 2022 and December 2024. Inclusion criteria: (1) Diagnosis of liver fibrosis confirmed by liver biopsy; (2) Completion of 2D-SWE ultrasound examination; (3) Interval between ultrasound and liver biopsy not exceeding 2 weeks. Exclusion criteria: (1) Patients with concurrent primary or metastatic liver tumors and/or splenic tumors confirmed by imaging or histology; (2) History of hepatectomy, liver transplantation, splenic embolization, or splenectomy; (3) Unsuccessful 2D-SWE measurements, defined as failure to obtain a stable elastogram with a color fill ratio >50% in the sampling frame after five attempts; (4) Incomplete clinical or pathological data essential for analysis. (5) Patients with other etiologies of liver fibrosis were excluded due to the small sample size (fewer than 10 cases per etiology).

**Figure 1:**
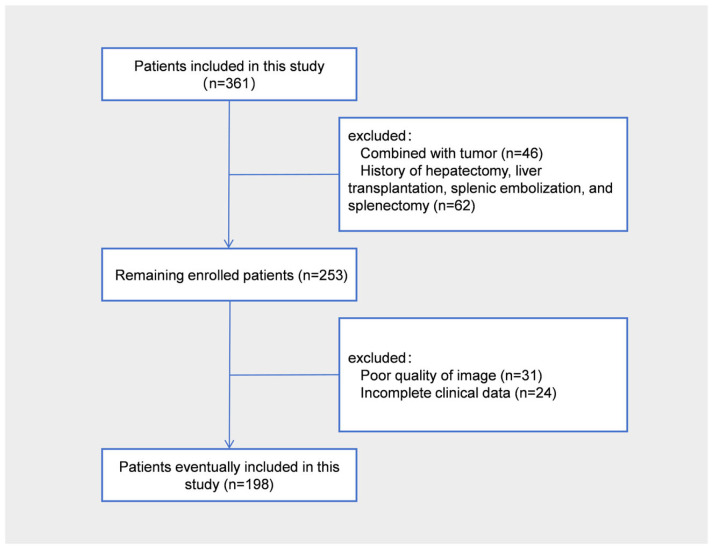
The results of patient enrollments. In total, 198 out of 361 patients were enrolled in this study.

The training and validation cohorts were randomly divided at a ratio of 7:3, with 136 patients finally included in the training cohort and 62 in the validation cohort (Figure 1).

### 2.2. Pathological Examination

Liver biopsy was performed on the right lobe of the liver using a 16-gauge or 18-gauge needle (BD, Franklin Lakes, NJ, USA), and all biopsy specimens were sent to the Department of Pathology of our hospital for examination. The specimens were fixed in 10% neutral buffered formalin (Thermo Fisher Scientific, Waltham, MA, USA), embedded in paraffin, sectioned, and subjected to HE staining, Masson’s trichrome staining, and reticular fiber staining. Fibrosis staging was conducted according to the Scheuer scoring system, which includes stages 1, 2, 3, and 4 [29].

### 2.3. Serological Examinations

Serological test results within one week after 2D-SWE were collected for each patient, including albumin (ALB), platelet count (PLT), aspartate aminotransferase (AST), alanine aminotransferase (ALT), gamma-glutamyl transpeptidase (GGT), total bilirubin (TBI), direct bilirubin (DBI), and indirect bilirubin (IBI). The noninvasive serum liver fibrosis indexes—APRI and FIB-4—were calculated [30,31]. The degree of hepatic steatosis in all patients was less than 5%, indicating no significant steatosis.

### 2.4. Two-Dimensional Shear Wave Elastography

A Supersonic Imagine Aixplorer ultrasonic diagnostic device equipped with an SC6-1 convex array probe (Supersonic Imagine, Aix-en-Provence, France; operating at a frequency range of 1–6 MHz) was utilized. Prior to the measurements, patients were required to undergo an 8-h fasting period. During the assessment of liver stiffness, participants were positioned in a supine posture, with their hands elevated above their heads to maximize intercostal space exposure. Initially, B-mode ultrasonic scanning was conducted, with a focus on minimizing interference from bile ducts and blood vessels. Once a distinct two-dimensional image was achieved, the scanning mode was transitioned to shear wave elastography (SWE). The 2D-SWE ROI was positioned 1–2 cm under the liver capsule, and a 1.2 cm diameter circular Q-Box ROI was placed in the 2D-SWE image. Patients were then directed to hold their breath, and the images were captured and saved once image stability was ensured and the color filled more than half of the sampling frame [32]. For each patient, five 2D-SWE images were acquired. The measurement protocol for the spleen replicated the methodology employed for the liver assessment.

### 2.5. Region-of-Interest Segmentation

**Figure 2:**
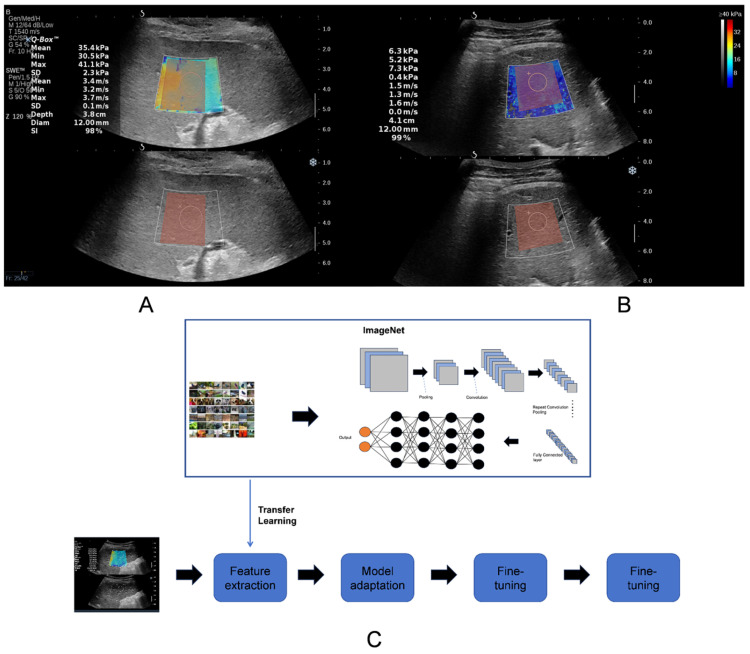
The ROI of radiomics (TL, ML) and the flow chart of transfer learning model in this study. (**A**) Spleen image. (**B**) Liver image. Image of elastogram image (**top**), gray scale image (**bottom**), stiffness measurement with Q-Box (white circle area), and ROI of radiomics (red square area). (**C**) Flow chart of transfer learning model.

The 2D-SWE images of the liver and spleen were imported using 3D-Slicer software (version 5.6.2, 3D Slicer Community, Boston, MA, USA). Two radiologists drew a square region of interest (ROI) as large as possible within the color-coded elastography trapezoidal frame, and the ROI was duplicated in the grayscale image below. Meanwhile, bile ducts and vascular tissues were avoided as much as possible (Figure 2).

### 2.6. Machine Learning

This study extracted radiomic features using the PyRadiomics tool (version 3.0.1, Radboud University Medical Center, Nijmegen, The Netherlands), including seven primary feature categories (e.g., first-order statistics, texture matrices, and 2D shape descriptors) and their derivative features generated via seven image transformations (e.g., Wavelet, Laplacian of Gaussian, and intensity adjustments). Only features with an intraclass correlation coefficient (ICC) > 0.8 (assessed for inter- and intra-observer reliability) were retained for subsequent analysis.

A three-step feature dimensionality reduction strategy was implemented to avoid overfitting: (1) non-parametric Kruskal–Wallis tests (*p* < 0.05) to filter features irrelevant to classification; (2) Pearson correlation analysis (|r| > 0.9) to eliminate multicollinearity; and (3) least absolute shrinkage and selection operator (LASSO) regression to identify the optimal feature subset.

To comprehensively evaluate the discriminative power of the handcrafted radiomic features and establish a robust performance benchmark, we developed and compared six conventional machine learning (ML) models based on the final features: logistic regression (LR), decision tree (DT), random forest (RF), support vector machine (SVM), gradient boosting machine (GBM), and extreme gradient boosting (XGB). This multi-model approach allowed us to assess the stability of the radiomic signatures across different algorithmic paradigms, from simple linear models (LR) to complex, non-linear ensemble methods (RF, GBM, XGB). Hyperparameters of all models were optimized using grid search cross-validation to ensure performance stability. The specific feature categories and parameter settings are detailed in the Supplementary File (File S1).

### 2.7. Transfer Learning

This research employed the ImageNet-pretrained EfficientNet-B4 as the backbone architecture; its efficacy had been confirmed in multiple organs such as the lungs, kidneys, and bone tissue in previous studies [33,34,35,36]. Additionally, we incorporated a custom classification head comprising Dropout layers (0.5 and 0.3 probability), linear transformations (reducing features to 512 dimensions), and ReLU activations for task-specific fine-tuning. To optimize input data quality, all images underwent a specialized preprocessing pipeline: mask application to isolate regions of interest, Gaussian blurring (5 × 5 kernel) for noise reduction, and LAB color space conversion with CLAHE enhancement (clip limit = 2.0, tile grid size = 8 × 8) to boost local contrast before resizing to standardized dimensions. Data augmentation techniques—including horizontal/vertical flipping (*p* = 0.5), random 90° rotation (*p* = 0.5), brightness/contrast variation (*p* = 0.2), and multi-parameter color jittering (brightness/contrast/saturation/hue at 0.2)—were systematically applied. These were integrated with WeightedRandomSampler to address class imbalance through inverse-frequency weighting, effectively expanding the training diversity while maintaining stratified data splits (train/val/test) based on label distributions. This comprehensive approach mitigated overfitting risks in limited-sample scenarios while optimizing feature discriminability for the target classification task.

### 2.8. Model Construction and Validation

We established machine learning and transfer learning models utilizing liver and spleen grayscale ultrasound images combined with 2D-SWE images to diagnose both liver fibrosis staging and etiology. Furthermore, the diagnostic performance of the radiomics models based on these ultrasound features was specifically compared with established biomarkers (APRI and FIB-4) in the assessment of liver fibrosis staging. The proposed deep learning framework in this study is centered on this transfer learning (TL) model, which served as our final classifier for both liver fibrosis staging and etiology diagnosis. Its performance was systematically compared against the six conventional ML models and serum biomarkers to validate its superiority.

### 2.9. Statistical Power and Multinomial Logistic Regression

To address the robustness of our findings, we conducted a post hoc statistical power analysis. The analysis was performed using Python software (version 3.11.6, Python Software Foundation, Wilmington, DE, USA), with an alpha level set at 0.05 and our study’s sample size (*n* = 198) as fixed parameters. The effect sizes (Cohen’s f) for each identified radiomic feature were calculated based on the observed variance explained in fibrosis staging. This analysis allows us to quantify the probability that our study correctly detected true effects, thereby directly responding to the concern regarding the strength of the results.

Furthermore, to complement the high-performance machine learning models and provide deeper insights into the non-linear relationships between radiomic features and multi-category fibrosis stages, we performed multinomial logistic regression (MLR) analysis. The MLR model included the top features selected by the LASSO algorithm, with polynomial terms (up to the second order) introduced for continuous variables to capture potential curvilinear effects. This complex regression analysis aims to validate the independent contribution of each feature across different stages and enhance the interpretability of our model.

### 2.10. Statistical Analysis

Descriptive statistical analyses were presented as mean ± standard deviation (SD) or median with interquartile range (IQR). For quantitative variables, comparisons were conducted using the *t*-test or Mann–Whitney U test, whereas categorical variables were analyzed via the chi-squared test or Fisher’s exact test. The receiver operating characteristic curve (ROC) area under the curve (AUC) served as the accuracy metric for evaluating diagnostic performance. Delong’s test was utilized to compare differences between AUC values. Sensitivity, specificity, positive predictive value (PPV), negative predictive value (NPV), and diagnostic likelihood ratios (LR+ and LR−) were calculated. All statistical analyses were performed using Python software (version 3.11.6, Python Software Foundation, Wilmington, DE, USA), with statistical significance defined as *p* < 0.05.

## 3. Results

### 3.1. Baseline Characters

Among the 198 patients enrolled in this study (age range 16–72 years), 136 comprised the training cohort and 62 constituted the validation cohort, which was utilized for diagnostic performance assessment of the model. Table 1 details the patients’ baseline data and clinical parameters, showing no statistically significant differences between the two cohorts on any variable (*p* > 0.05).

### 3.2. Radiomic Feature Selection

We constructed Lasso models for four ultrasound modalities—liver grayscale, liver 2D-SWE, spleen grayscale, and spleen 2D-SWE—and visualized feature contributions using SHAP summary plots for both liver fibrosis staging and etiology diagnosis. The analysis focused on feature importance and type distribution.

**Figure 3:**
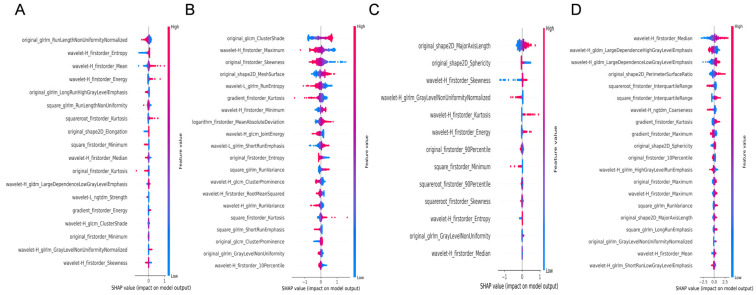
SHAP feature importance analysis of the LASSO regression model for liver fibrosis staging. (**A**,**B**): Feature importance visualization for hepatic B-mode and 2D-SWE images, respectively. (**C**,**D**): Corresponding analyses for splenic B-mode and 2D-SWE images.

**Figure 4:**
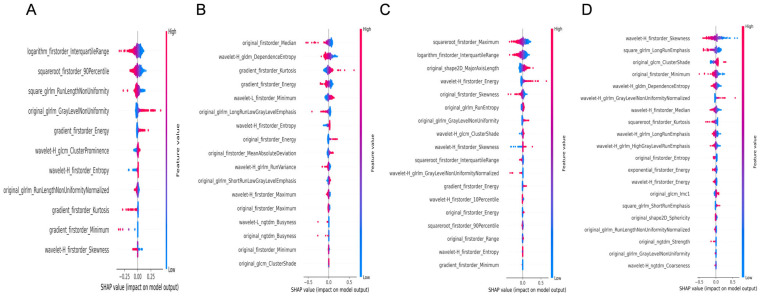
SHAP feature importance analysis of the LASSO regression model for etiology diagnosis. (**A**,**B**): Feature importance visualization for hepatic B-mode and 2D-SWE images, respectively. (**C**,**D**): Corresponding analyses for splenic B-mode and 2D-SWE images.

1. For liver fibrosis staging (Figure 3):
  1.1 Liver grayscale images prioritized Gray-Level Run-Length Matrix (GLRLM) texture and wavelet-transformed first-order statistics.
  1.2 Liver 2D-SWE emphasized Gray-Level Co-occurrence Matrix (GLCM) texture and first-order statistics.
  1.3 Spleen grayscale imaging prioritized shape features (original_shape2D_Major-AxisLength and original_shape2D_Sphericity).
  1.4 Spleen 2D-SWE focused on wavelet-transformed first-order statistics and Gray-Level Dependence Matrix (GLDM) texture.

2. For etiology diagnosis (Figure 4):
  2.1 Liver grayscale images prioritized distributional statistics and texture heterogeneity, logarithm_firstorder_InterquartileRange and squareroot_firstorder_90Perce-ntile capturing asymmetric or focal damage patterns.
  2.2 Liver 2D-SWE emphasized stiffness distribution and complexity (original_firstorder_Median and wavelet-H_gldm_DependenceEntropy), distinguishing etiologies by stiffness homogeneity/heterogeneity.
  2.3 Spleen grayscale imaging integrated morphological and distributional features (original_shape2D_MajorAxisLength and squareroot_firstorder_Maximum).
  2.4 Spleen 2D-SWE focused on asymmetric stiffness and local texture (wavelet-H_firstorder_Skewness and square_glrlm_LongRunEmphasis).

### 3.3. Diagnostic Accuracy of Liver Fibrosis Staging

The diagnostic performance of six ML models (LR, DT, RF, SVM, GBM, XGB) and TL models for liver fibrosis staging was evaluated across four imaging modalities: liver grayscale, spleen grayscale, liver 2D-SWE, and spleen 2D-SWE.

For liver 2D-SWE images, SVM, GBM, XGB, and RF exhibited exceptional validation performance, with AUC ≥0.98 for S4, ≥0.98 for ≥S3, and ≥0.99 for ≥S2, accompanied by sensitivity and specificity both exceeding 0.88 (*p* < 0.001, Supplementary Table S1 and Figure S1). Similarly, spleen 2D-SWE images also achieved excellent results. SVM, GBM, and XGB showed AUC ≥0.97 for S4, ≥0.97 for ≥S3, and ≥0.99 for ≥S2 (*p* < 0.001, Supplementary Table S2 and Figure S2). In contrast, grayscale images (liver and spleen) had lower discriminative power, though RF, GBM, and XGB still outperformed LR and DT. For liver grayscale images, RF (AUC 0.92 for S4, 0.88 for ≥S3, 0.81 for ≥S2, *p* < 0.01 or *p* < 0.001) performed best. For spleen grayscale images, GBM achieved the highest diagnostic accuracy (AUC 0.92 for S4, 0.87 for ≥S3, 0.82 for ≥S2) (*p* < 0.001, Supplementary Tables S3 and S4 and Figures S3 and S4).

Among models, 2D-SWE-based models consistently outperformed grayscale-based models. LR and DT consistently performed the poorest across modalities and stages, while tree-based ensembles (RF, GBM, XGB) and SVM maintained robustness in validation cohorts. Additionally, model discriminative ability declined from S4 to ≥S2 in grayscale images but remained high in 2D-SWE, which proved 2D-SWE’s superiority. Training cohorts often showed near-perfect performance (AUC ≥ 0.99) for most models, but validation results revealed overfitting in DT and LR.

**Figure 5:**
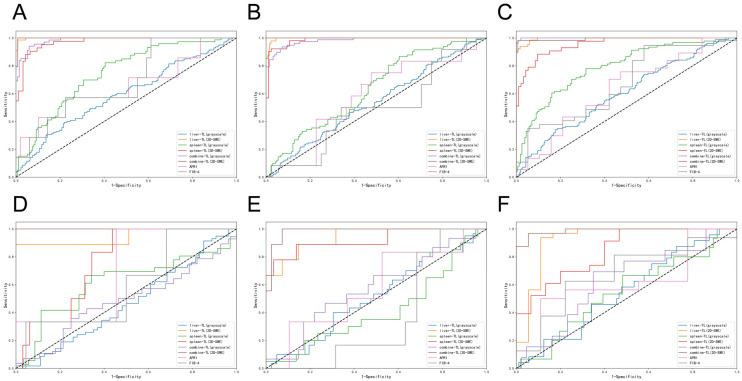
Comparison of ROC curves between TL and biomarkers for the assessment of liver fibrosis staging in training and validation cohorts (**A**,**D**): S0–S1 versus S2–S4 (≥S2) in training and validation cohorts; (**B**,**E**): S0–S2 versus S3–S4 (≥S3) in training and validation cohorts; (**C**,**F**): S0–S3 versus S4 (S4) in training and validation cohorts.

TL models of different modalities exhibit significant differences in performance. In grayscale images, their performance was limited. Liver grayscale TL achieved AUC of 0.60, 0.55 for S4; 0.54, 0.51 for ≥S3; 0.59, 0.46 for ≥S2 in training and validation cohorts. Spleen grayscale TL had validation AUC of 0.55 (S4), 0.42 (≥S3), and 0.61 (≥S2). Liver–spleen combined TL (CTL) in grayscale also performed poorly (S4: 0.62, ≥S3: 0.57, ≥S2: 0.48). Nevertheless, in 2D-SWE images, TL improved markedly. Liver TL had training AUC 0.99 for all stages and validation AUC 0.92 (S4), 0.94 (≥S3 and ≥S2). In comparison with liver TL, the diagnostic performance of spleen TL showed a decline, with the best performance observed in the ≥S3 stage (validation AUC 0.92). Notably, CTL in 2D-SWE showed exceptional validation performance, achieving AUCs of 0.99 (with 0.84 sensitivity and 1.00 specificity) for S4, 0.98 (with 1.00 sensitivity and 0.90 specificity) for ≥S3, and 1.00 (with 1.00 sensitivity and 0.98 specificity) for ≥S2, highlighting the value of multi-organ integration (*p* < 0.001, Table 2 and Figure 5).

APRI and FIB-4 exhibited poor performance across all stages. APRI had validation AUC of 0.62 (S4), 0.58 (≥S3), and 0.70 (≥S2). FIB-4 performed even worse, with validation AUC of 0.67 (S4), 0.37 (≥S3), and 0.59 (≥S2), further confirming the superiority of radiomics models, particularly 2D-SWE-based ones (*p* < 0.001, Table 2 and Figure 5).

### 3.4. Diagnostic Accuracy of Liver Fibrosis Etiology

Based on liver grayscale images, all ML models (RF, DT, LR, SVM, GBM, XGB) demonstrated perfect performance on the training cohort (AUC = 0.99–1.00). However, performance significantly declined in the validation cohort. RF achieved the highest validation AUC (0.78, 95% CI: 0.72–0.85), while DT performed poorest (AUC = 0.56, 95% CI: 0.46–0.66, *p* < 0.001). The TL model showed modest performance (Validation AUC = 0.73, 95% CI: 0.64–0.81). Spleen models were similar to the liver, ML models exhibited excellent training performance (AUC ≥ 0.80) but substantial validation drops. GBM achieved the highest validation AUC (0.81, 95% CI: 0.73–0.88), while TL performed poorest (Validation AUC = 0.57, 95% CI: 0.44–0.68, *p* < 0.001). The CTL model performed comparably to the best ML models (Validation AUC = 0.79, 95% CI: 0.73–0.86, *p* < 0.01). However, a pronounced performance gap between training and validation cohorts was evident for most ML models, indicating potential overfitting (Table 3, Supplementary Figure S5).

**Figure 6:**
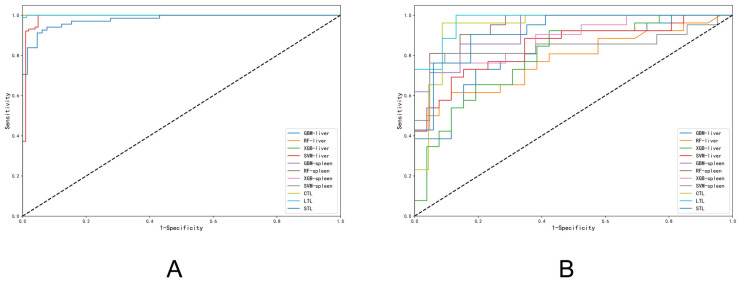
Comparison of ROC curves between ML and TL for etiology diagnosis using grayscale images in training and validation cohorts (**A**): training cohort. (**B**): validation cohort. CTL, combined transfer learning; LTL: liver transfer learning; STL: spleen transfer learning; RF, random forest; SVM, support vector machine; GBM, gradient boosting machine; XGB, extreme gradient boosting.

In liver 2D-SWE images, ML models again showed strong training performance. Validation performance was generally superior to grayscale models. SVM (Validation AUC = 0.84, 95% CI: 0.73–0.95) and GBM (Validation AUC = 0.82, 95% CI: 0.70–0.93) were the top ML performers (*p* < 0.001). Crucially, the TL model demonstrated exceptional performance, achieving the highest validation AUC (0.97, 95% CI: 0.93–1.00, *p* < 0.001) with balanced sensitivity (87%) and specificity (92%). In spleen models, RF (Validation AUC = 0.94, 95% CI: 0.88–1.00), GBM (Validation AUC = 0.93, 95% CI: 0.86–0.99), XGB (Validation AUC = 0.88, 95% CI: 0.77–0.98), and TL (Validation AUC = 0.92, 95% CI: 0.82–0.99) all showed high discriminatory ability. The CTL model achieved excellent validation performance (AUC = 0.94, 95% CI: 0.85–1.00; Specificity = 96%, PPV = 95%, *p* < 0.001). Notably, while a training-validation gap persisted, its magnitude was generally smaller compared to grayscale models, suggesting better generalization with 2D-SWE features (Table 4 and Figure 6).

### 3.5. Assessment of Statistical Power and Multinomial Regression Results

**Figure 7:**
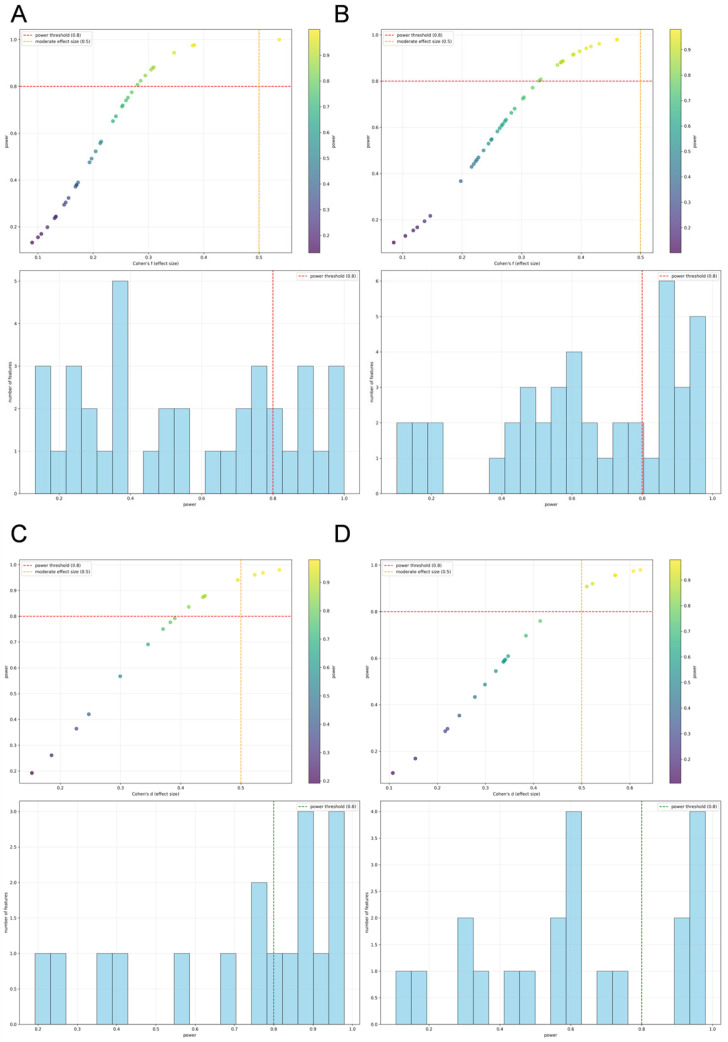
Post hoc Power Analysis for Liver Fibrosis Staging and Etiology Classification Based on Radiomic Features (**A**,**B**): Statistical power as a function of effect size for classifying fibrosis stages using liver and spleen radiomic features, respectively. The histogram displays the distribution of observed effect sizes across all features. (**C**,**D**): Corresponding power analysis for the classification of fibrosis etiology based on liver and spleen features.

The post hoc power analysis, summarized in Figure 7, demonstrates that our study possesses sufficient statistical power for the majority of the key radiomic features identified. The distribution of power values indicates that a significant proportion of features achieved a statistical power exceeding the conventional threshold of 0.8. This confirms that the study was adequately powered to detect clinically relevant effects, strengthening the validity of the primary findings related to these robust features.

**Figure 8:**
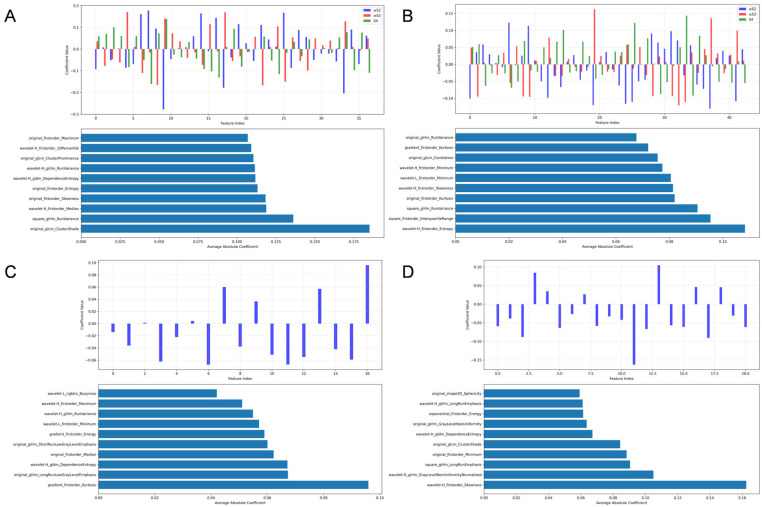
Multinomial Logistic Regression Analysis of Radiomic Features for Liver Fibrosis Staging and Etiology Classification (**A**,**B**): Coefficients of the multinomial logistic regression model for discriminating fibrosis stages based on liver and spleen radiomic features, respectively. For each panel, the upper bar plotvisualizes the coefficient values for each feature across different outcome categories, the lower bar plotranks the features by the sum of the absolute values of their coefficients, highlighting the most discriminative features overall. (**C**,**D**): Corresponding analysis for the classification of fibrosis etiology based on liver and spleen features.

The multinomial logistic regression analysis corroborated the importance of the spleen- and liver-derived radiomic features. The regression coefficients revealed distinct, stage-specific contribution patterns. The inclusion of polynomial terms significantly improved the model fit for several features, suggesting the presence of non-linear relationships that are effectively captured by our machine learning models. The consistency between the MLR results and the feature importance rankings from the ML and TL models reinforces the credibility of the identified biomarkers (Figure 8).

## 4. Discussion

This study establishes that radiomics models leveraging 2D-SWE of both liver and spleen significantly advance the non-invasive diagnosis of liver fibrosis. By integrating multi-organ imaging with transfer learning techniques, we achieved exceptional accuracy in fibrosis staging and etiology differentiation, outperforming conventional serum biomarkers (APRI, FIB-4) and grayscale-based radiomics, this is consistent with previous research findings [21,24,37]. These findings underscore the transformative potential of quantitative elastography features in capturing microstructural alterations induced by fibrotic remodeling, which remain invisible to traditional ultrasound or biochemical indices.

The superiority of 2D-SWE radiomics is rooted in its ability to quantify tissue stiffness heterogeneity with high spatial resolution. While prior studies validated 2D-SWE as a reliable tool for fibrosis assessment [8,9,38,39], our work demonstrates that radiomic feature extraction from elastography images substantially enhances diagnostic precision beyond simple stiffness measurements. For instance, in liver fibrosis staging, GLCM textures from 2D-SWE data were critical discriminators, reflecting subtle variations in collagen distribution and architectural distortion across fibrosis stages. Gray-level textures represent an advanced statistical approach for quantifying the spatial distribution of gray-level pixel intensities within a localized area. Numerous studies have confirmed that the GLCM can help infer the underlying organization and structure of collagen fibers, and it has been applied to the diagnosis of liver fibrosis [40,41,42,43,44]. This explains why machine learning models (such as SVM, GBM) using 2D-SWE inputs consistently achieved near-perfect validation AUC ≥ 0.97.

A pivotal innovation lies in the integration of splenic radiomics. Pathophysiologically, splenic enlargement (captured by features like original_shape2D_MajorAxisLength) and altered stiffness patterns are direct consequences of portal hypertension, which correlates with advanced fibrosis [45,46,47]. Our data revealed that splenic 2D-SWE features, particularly wavelet-H_firstorder_Skewness and GLDM textures are complemented hepatic data by encoding portal hemodynamic changes. This multi-organ approach proved most potent in the combined liver–spleen transfer learning (TL) model, which achieved 100% sensitivity and 98% specificity for ≥S2 fibrosis in validation. Such performance transcends previous radiomics studies focused solely on the liver and aligns with the clinical paradigm that considers splenic metrics in portal hypertension assessment.

For etiology diagnosis, transfer learning emerged as a breakthrough. The TL model using liver 2D-SWE attained a validation AUC of 0.97 in distinguishing hepatitis B virus (HBV) from autoimmune liver diseases (AILD), significantly outperforming handcrafted ML models. We posit that pre-trained convolutional neural networks (CNNs) excel at identifying etiology-specific stiffness patterns: chronic HBV typically exhibits homogeneous stiffness increases due to diffuse fibrosis, while AILD often shows heterogeneous stiffness from necroinflammatory activity, ranging from spotty to confluent necrosis [48,49]. SHAP analysis (Figure 4B) further confirmed this observation, with key discriminative features including median stiffness (original_firstorder_Median) and entropy-based heterogeneity (wavelet-H_gldm_DependenceEntropy). These patterns evade manual feature engineering but are learnable by deep networks through hierarchical representation learning. This capability addresses a critical unmet need, as no prior studies have systematically applied deep learning to differentiate fibrosis etiologies using ultrasound.

Notably, in some tasks such as etiological diagnosis based on grayscale images, the diagnostic performance of ML is similar to that of TL, and ML even outperforms TL. We believe this proves that the handcrafted radiomic features extracted from grayscale images themselves possess a certain degree of discriminative ability. However, the obvious performance gap between the training set and validation set of most ML models indicates severe overfitting, meaning the baseline models perform poorly under this mode. In our view, this overfitting does not stem from the algorithms themselves, but from the inherent limitations of grayscale ultrasound features, which provide limited discriminative ability and poor reproducibility [50,51,52]. This is a challenge faced by almost all models, including both ML and TL models. This shows that the primary factor driving performance improvement is high-quality imaging data (2D-SWE). When we switch to 2D-SWE images with richer information, the performance of all models achieves a qualitative leap. This also happens to prove that the key advantage of TL lies in its superior robustness and generalization. Even based on grayscale images, we can still observe a consistently small performance drop between the training set and validation set. Therefore, when applied to information-rich 2D-SWE data, the value of TL becomes even more prominent.

Despite these advances, limitations warrant consideration. First, the retrospective design and moderate cohort size (*n* = 198) necessitate validation in larger, prospective multi-center cohorts. Second, restricting etiologies to HBV and AILD limits generalizability; future studies should incorporate prevalent causes like metabolic dysfunction-associated steatotic liver disease (MASLD) and Hepatitis C virus (HCV). Third, manual ROI placement introduces operator dependency, but this may be mitigated by emerging auto-segmentation algorithms. Finally, the model itself also requires further optimization to more comprehensively integrate other clinical data, such as serological results, in order to improve the overall performance of the model, reduce the problem of overfitting, and achieve more accurate non-invasive diagnosis of liver fibrosis staging and etiology.

We anticipate that deep learning may achieve further advancements in the diagnosis of liver diseases. MicroRNAs (miRNAs), as a class of small non-coding endogenous RNA molecules, regulate gene expression through translational repression or mRNA degradation. They can modulate the activation and polarization of macrophages, thereby inducing liver fibrosis [53,54]. A number of studies have combined deep learning frameworks with molecular technologies such as miRNA, yielding promising results [55,56,57]. Therefore, in future research, radiomics can be integrated with genomics, molecular omics, and other omics disciplines to form a multiomics and multimodal diagnostic framework. This framework will address the limitations of traditional methods and is expected to become a novel diagnostic technology.

## 5. Conclusions

This study develops a novel deep learning framework that integrates liver–spleen 2D-SWE radiomics to achieve accurate liver fibrosis staging and etiology diagnosis, demonstrating superior diagnostic accuracy compared to existing biomarkers and single-modality approaches. These findings highlight the potential of multi-organ imaging radiomics in advancing precision hepatology. Future studies should prioritize clinical validation to facilitate its integration into clinical workflows.

## Figures and Tables

**Table 1:** Baseline characteristics of patients.

Variable	All Patients	Training Cohort	Validation Cohort	*p*-Value
Number of patients	198 (100.0%)	136 (68.7%)	62 (31.3%)	-
Gender (male)	117(59.1%)	82(60.3%)	35(56.5%)	0.610
Age (years)	51.1 ± 15.6	50.3 ± 16.3	52.9 ± 14.5	0.624
SD (mm)	145.2 ± 36.7	140.6 ± 33.8	156.5 ± 42.3	0.210
ST (mm)	49.8 ± 14.0	47.8 ± 12.7	54.7 ± 16.2	0.154
SVD (mm)	9.6 ± 3.9	9.5 ± 3.8	9.9 ± 4.4	0.770
PVD (mm)	12.8 ± 2.8	13.0 ± 3.0	12.4 ± 2.4	0.535
PVV (cm/s)	25.3 ± 10.4	25.2 ± 10.8	25.6 ± 9.8	0.925
AST (U/L)	43.6 ± 33.2	40.9 ± 36.1	49.8 ± 25.3	0.425
ALT (U/L)	34.5 ± 31.3	35.3 ± 35.9	32.7 ± 17.7	0.808
GGT (U/L)	83.6 ±112.1	64.7 ± 67.7	127.1 ± 173.0	0.094
ALB (g/L)	38.4 ± 5.8	39.1 ± 6.2	36.8 ± 4.7	0.254
TBIL (μmol/L)	35.4 ± 33.1	34.9 ± 36.1	36.6 ± 26.3	0.878
DBIL (μmol/L)	11.4 ± 16.8	12.6 ± 19.7	8.7 ± 5.8	0.493
IBIL (μmol/L)	24.1 ± 19.5	22.4 ± 18.5	28.1 ± 21.8	0.387
PLT (10^9^/L)	126.9 ±72.5	134.0 ± 76.1	110.6 ± 63.4	0.338
Etiology				0.295
HBV	101 (51.0%)	69 (50.7%)	32 (51.6%)	
AILD	97 (49.0%)	67 (49.3%)	30 (48.4%)	
Fibrosis stages				0.997
S2	58 (29.3%)	40 (29.4%)	18 (29.0%)	
S3	51 (25.8%)	35 (25.7%)	16 (25.8%)	
S4	89 (44.9%)	61 (44.9%)	28 (45.2%)	

ALT: Alanine aminotransferase; ALB: Albumin; AST: Aspartate aminotransferase; AILD: Autoimmune Liver Diseases; DBIL: Direct bilirubin; GGT: Gamma-glutamyl transpeptidase; HBV: Hepatitis B virus; IBIL: Indirect bilirubin; PVD: Portal vein diameter; PVV: Portal vein flow velocity; PLT: Platelet; ST: Splenic thickness; SVD: Splenic vein diameter; SD: Spleen diameter; TBIL: Total bilirubin.

**Table 2:** Performance of TL models and biomarker models for liver fibrosis staging in training and validation cohorts.

		AUC	Sensitivity (%)	Specificity (%)	PPV (%)	NPV (%)	LR+	LR−
liver grayscale images								
S4								
TL	T	0.60 (0.55–0.66)	0.16	0.92	0.49	0.71	2.10	0.91
	V	0.55 (0.43–0.67)	0.13	0.93	0.33	0.80	1.83	0.94
≥S3								
TL	T	0.54 (0.48–0.60)	0.34	0.75	0.42	0.69	1.37	0.88
	V	0.51 (0.39–0.63)	0.33	0.72	0.30	0.75	1.19	0.93
≥S2								
TL	T	0.59 (0.54–0.65)	0.69	0.42	0.39	0.72	1.20	0.73
	V	0.46 (0.35–0.58)	0.62	0.37	0.51	0.48	0.99	1.02
spleen grayscale images								
S4								
TL	T	0.79 (0.72–0.84)	0.76	0.68	0.58	0.83	2.35	0.36
	V	0.55 (0.38–0.71)	0.47	0.64	0.26	0.82	1.31	0.83
≥S3								
TL	T	0.63 (0.56–0.70)	0.24	0.84	0.43	0.69	1.54	0.90
	V	0.42 (0.26–0.57)	0.20	0.92	0.50	0.75	2.56	0.87
≥S2								
TL	T	0.75 (0.68–0.81)	0.55	0.76	0.49	0.80	2.31	0.59
	V	0.61 (0.48–0.75)	0.69	0.69	0.69	0.68	2.21	0.45
liver 2D-SWE images								
S4								
TL	T	0.99 (0.99–1.00)	0.83	1.00	1.00	0.91	-	0.17
	V	0.92 (0.81–1.00)	0.84	0.89	0.93	0.76	7.59	0.18
≥S3								
TL	T	0.99 (0.99–1.00)	1.00	0.94	0.85	1.00	16.63	0.00
	V	0.94 (0.84–1.00)	0.78	0.93	0.70	0.95	10.63	0.24
≥S2								
TL	T	0.99 (0.99–1.00)	1.00	0.97	0.96	1.00	37.33	0.00
	V	0.94 (0.79–1.00)	0.89	0.93	0.73	0.97	12.15	0.12
spleen 2D-SWE images								
S4								
TL	T	0.95 (0.92–0.98)	0.84	0.89	0.78	0.92	7.79	0.18
	V	0.84 (0.70–0.95)	0.74	0.73	0.81	0.65	2.78	0.36
≥S3								
TL	T	0.99 (0.97–1.00)	0.88	0.99	0.98	0.93	75.00	0.12
	V	0.92 (0.78–1.00)	0.78	0.97	0.88	0.93	22.56	0.23
≥S2								
TL	T	0.97 (0.94–0.99)	0.90	0.94	0.86	0.96	14.17	0.10
	V	0.76 (0.57–0.91)	0.33	0.78	0.22	0.86	1.52	0.85
CTL(grayscale)								
S4								
	T	0.99 (0.99–1.00)	0.98	0.98	0.95	0.99	47.47	0.02
	V	0.62 (0.45–0.80)	0.38	0.74	0.31	0.80	1.50	0.83
≥S3								
	T	0.98 (0.96–0.99)	0.96	0.87	0.82	0.97	7.45	0.04
	V	0.57 (0.39–0.75)	0.47	0.68	0.35	0.78	1.47	0.78
≥S2								
	T	0.98 (0.97–0.99)	0.75	0.99	0.96	0.89	49.88	0.25
	V	0.48 (0.33–0.63)	0.39	0.68	0.55	0.53	1.22	0.89
CTL(2D-SWE)								
S4								
	T	0.99 (0.98–1.00)	0.93	1.00	1.00	0.97	-	0.07
	V	0.99 (0.96–1.00)	0.84	1.00	1.00	0.78	-	0.16
≥S3								
	T	1.00 (1.00–1.00)	1.00	0.97	0.96	1.00	37.67	0.00
	V	0.98 (0.95–1.00)	1.00	0.90	0.69	1.00	10.25	0.00
≥S2								
	T	0.99 (0.99–1.00)	1.00	0.99	0.98	1.00	122.00	0.00
	V	1.00 (1.00–1.00)	1.00	0.98	0.90	1.00	41.00	0.00
APRI								
S4								
	T	0.63 (0.48–0.78)	0.38	0.79	0.78	0.39	1.80	0.79
	V	0.62 (0.39–0.85)	0.50	0.89	0.89	0.50	4.50	0.56
≥S3								
	T	0.58 (0.39–0.77)	0.17	0.91	0.33	0.80	1.83	0.92
	V	0.58 (0.31–0.85)	0.33	0.89	0.50	0.81	3.17	0.75
≥S2								
	T	0.65 (0.42–0.89)	0.71	0.45	0.16	0.92	1.30	0.64
	V	0.70 (0.35–1.00)	0.67	0.55	0.17	0.92	1.47	0.61
FIB-4								
S4								
	T	0.67 (0.52–0.81)	0.92	0.42	0.76	0.73	1.59	0.19
	V	0.67 (0.42–0.89)	0.88	0.22	0.67	0.50	1.12	0.56
≥S3								
	T	0.49 (0.31–0.67)	0.42	0.66	0.25	0.81	1.22	0.89
	V	0.37 (0.16–0.59)	0.17	0.58	0.11	0.69	0.40	1.44
≥S2								
	T	0.69 (0.44–0.90)	0.86	0.37	0.17	0.95	1.40	0.37
	V	0.59 (0.25–1.00)	1.00	0.32	0.17	1.00	1.47	0.00

AUC, area under the receiver operating characteristic curve; LR+, positive diagnostic likelihood ratio; LR−, negative diagnostic likelihood ratio; NPV, negative predictive value; PPV, positive predictive value; TL, transfer learning; CTL, combined transfer learning; T, training cohort; V, validation cohort.

**Table 3:** Performance of radiomics models (ML,TL) for etiology diagnosis using grayscale images in training and validation cohorts.

		AUC	Sensitivity (%)	Specificity (%)	PPV (%)	NPV (%)	LR+	LR−
Liver								
RF	T	1.00(1.00–1.00)	1.00	1.00	1.00	1.00	-	0.00
	V	0.78(0.72–0.85)	0.71	0.70	0.70	0.70	2.33	0.42
DT	T	0.99(0.99–1.00)	0.98	0.99	0.99	0.98	389.00	0.02
	V	0.56(0.46–0.66)	0.54	0.57	0.55	0.55	1.23	0.82
LR	T	0.66(0.63–0.70)	0.62	0.65	0.64	0.63	1.74	0.59
	V	0.69(0.61–0.76)	0.61	0.68	0.65	0.63	1.88	0.58
SVM	T	0.80(0.77–0.83)	0.76	0.73	0.74	0.75	2.78	0.33
	V	0.76(0.79–0.83)	0.68	0.68	0.68	0.68	2.09	0.48
GBM	T	1.00(1.00–1.00)	1.00	1.00	1.00	1.00	-	0.00
	V	0.76(0.70–0.83)	0.72	0.70	0.70	0.71	2.37	0.41
XGB	T	1.00(1.00–1.00)	1.00	1.00	1.00	1.00	-	0.00
	V	0.76(0.70–0.83)	0.68	0.70	0.69	0.68	2.23	0.46
TL	T	0.76(0.72–0.80)	0.52	0.82	0.73	0.64	2.82	0.59
	V	0.73(0.64–0.81)	0.48	0.81	0.62	0.71	2.52	0.64
Spleen								
RF	T	1.00(1.00–1.00)	1.00	1.00	1.00	1.00	-	0.00
	V	0.78(0.70–0.86)	0.65	0.73	0.71	0.68	2.44	0.48
DT	T	1.00(0.99–1.00)	0.99	1.00	1.00	0.99	-	0.01
	V	0.61(0.47–0.74)	0.57	0.63	0.61	0.59	1.55	0.68
LR	T	0.80(0.76–0.84)	0.73	0.69	0.70	0.72	2.35	0.40
	V	0.68(0.59–0.78)	0.65	0.55	0.59	0.61	1.44	0.64
SVM	T	0.99(0.99–1.00)	0.95	0.98	0.98	0.95	45.40	0.06
	V	0.78(0.70–0.87)	0.62	0.82	0.77	0.68	3.36	0.47
GBM	T	1.00(1.00–1.00)	1.00	1.00	1.00	1.00	-	0.00
	V	0.81(0.73–0.88)	0.70	0.78	0.76	0.72	3.23	0.38
XGB	T	1.00(1.00–1.00)	1.00	1.00	1.00	1.00	-	0.00
	V	0.74(0.66–0.83)	0.63	0.73	0.70	0.67	2.38	0.50
TL	T	0.72(0.66–0.77)	0.45	0.82	0.71	0.61	2.56	0.66
	V	0.57(0.44–0.68)	0.36	0.83	0.58	0.67	2.15	0.77
Combined- TL	T	0.96(0.94–0.98)	0.78	0.97	0.94	0.87	22.64	0.23
	V	0.79(0.73–0.86)	0.61	0.82	0.68	0.76	3.35	0.48

AUC, area under the receiver operating characteristic curve; LR+, positive diagnostic likelihood ratio; LR−, negative diagnostic likelihood ratio; NPV, negative predictive value; PPV, positive predictive value; TL, transfer learning; Combined-TL, combined transfer learning; T, training cohort; V, validation cohort; LR, logistic regression; DT, decision tree; RF, random forest; SVM, support vector machine; GBM, gradient boosting machine; XGB, extreme gradient boosting.

**Table 4:** Performance of radiomics models (ML,TL) for etiology diagnosis using 2D-SWE images in training and validation cohorts.

		AUC	Sensitivity (%)	Specificity (%)	PPV (%)	NPV (%)	LR+	LR−
Liver								
RF	T	1.00(1.00–1.00)	1.00	1.00	1.00	1.00	-	0.00
	V	0.78(0.65–0.91)	0.62	0.81	0.76	0.68	3.20	0.48
DT	T	0.99(0.99–1.00)	0.98	0.99	0.99	0.98	389.00	0.02
	V	0.77(0.58–0.96)	0.62	0.81	0.76	0.68	3.20	0.48
LR	T	0.78(0.71–0.84)	0.62	0.65	0.64	0.63	1.74	0.59
	V	0.72(0.58–0.86)	0.62	0.73	0.70	0.66	2.29	0.53
SVM	T	0.99(0.98–1.00)	0.76	0.73	0.74	0.75	2.78	0.33
	V	0.84(0.73–0.95)	0.73	0.77	0.76	0.74	3.17	0.35
GBM	T	1.00(1.00–1.00)	1.00	1.00	1.00	1.00	-	0.00
	V	0.82(0.70–0.93)	0.65	0.81	0.77	0.70	3.40	0.43
XGB	T	1.00(1.00–1.00)	1.00	1.00	1.00	1.00	-	0.00
	V	0.79(0.67–0.92)	0.65	0.73	0.71	0.68	2.43	0.47
TL	T	0.99(0.99–1.00)	0.99	0.99	0.99	0.99	94.75	0.01
	V	0.97(0.93–1.00)	0.87	0.92	0.91	0.89	11.30	0.14
Spleen								
RF	T	1.00(1.00–1.00)	1.00	1.00	1.00	1.00	-	0.00
	V	0.94(0.88–1.00)	0.71	0.95	0.94	0.77	15.00	0.30
DT	T	1.00(1.00–1.00)	1.00	1.00	1.00	1.00	-	0.00
	V	0.83(0.62–1.00)	0.76	0.90	0.89	0.79	8.00	0.26
LR	T	0.93(0.89–0.96)	0.80	0.87	0.86	0.81	6.09	0.23
	V	0.81(0.68–0.94)	0.71	0.76	0.75	0.73	3.00	0.38
SVM	T	1.00(1.00–1.00)	0.99	1.00	1.00	0.99	-	0.01
	V	0.84(0.70–0.98)	0.67	0.95	0.93	0.74	14.00	0.35
GBM	T	1.00(1.00–1.00)	1.00	1.00	1.00	1.00	-	0.00
	V	0.93(0.86–0.99)	0.71	0.86	0.83	0.75	5.00	0.33
XGB	T	1.00(1.00–1.00)	1.00	1.00	1.00	1.00	-	0.00
	V	0.88(0.77–0.98)	0.76	0.90	0.89	0.79	8.00	0.26
TL	T	0.98(0.96–0.99)	0.94	0.91	0.91	0.94	10.64	0.07
	V	0.92(0.82–0.99)	0.82	0.86	0.82	0.86	5.76	0.21
Combined- TL	T	1.00(1.00–1.00)	0.98	1.00	1.00	0.98	-	0.03
	V	0.94(0.85–1.00)	0.78	0.96	0.95	0.83	20.35	0.23

AUC, area under the receiver operating characteristic curve; LR+, positive diagnostic likelihood ratio; LR−, negative diagnostic likelihood ratio; NPV, negative predictive value; PPV, positive predictive value; TL, transfer learning; Combined-TL, combined transfer learning; T, training cohort; V, validation cohort; LR, logistic regression; DT, decision tree; RF, random forest; SVM, support vector machine; GBM, gradient boosting machine; XGB, extreme gradient boosting.

## Data Availability

The datasets presented in this article are not readily available due to patient privacy concerns. Requests to access the datasets should be directed to the First Hospital of Lanzhou University.

## Supplementary Materials

The following supporting information can be downloaded at: https://www.mdpi.com/article/10.3390/diagnostics15232986/s1, File S1: Settings of Machine Learning Parameters; Figure S1: Comparison of ROC curves between ML and TL for liver fibrosis staging using liver 2D-SWE images in training and validation cohorts; Figure S2: Comparison of ROC curves between ML and TL for liver fibrosis staging using spleen 2D-SWE images in training and validation cohorts; Figure S3: Comparison of ROC curves between ML and TL for liver fibrosis staging using liver grayscale images in training and validation cohorts; Figure S4: Comparison of ROC curves between ML and TL for liver fibrosis staging using spleen grayscale images in training and validation cohorts; Figure S5: Comparison of ROC curves between ML and TL for etiology diagnosis using grayscale images in training and validation cohorts; Table S1: Performance of machine learning models for liver fibrosis staging using liver 2D-SWE images in training and validation cohorts; Table S2: Performance of machine learning models for liver fibrosis staging using spleen 2D-SWE images in training and validation cohorts; Table S3: Performance of machine learning models for liver fibrosis staging using liver grayscale images in training and validation cohorts; Table S4: Performance of machine learning models for liver fibrosis staging using spleen grayscale images in training and validation cohorts.
