# Supplementary material for: A Novel Deep Learning Framework for Liver Fibrosis Staging and Etiology Diagnosis Using Integrated Liver–Spleen Elastography
Source: Diagnostics (Basel). 2025 Nov 24;15(23):2986. doi: 10.3390/diagnostics15232986 (PMC12691310; doi:10.3390/diagnostics15232986)
Supplement: Supplementary file 1 [file diagnostics-15-02986-s001.zip › diagnostics-3982240-supplementary.pdf]

File S1

1. Detailed Machine Learning Feature Extraction Parameters (PyRadiomics)

1.1 Primary Feature Categories and Spatial Configurations

Feature Category	Specific Parameters
Gray-Level Co-occurrence Matrix (GLCM)	Adjacency distance: [1]; Angular orientations: [0°, 45°, 90°, 135°]
Gray-Level Run-Length Matrix (GLRLM)	Distance: [1]; Angles: [0°, 45°, 90°, 135°]
Gray-Level Size Zone Matrix (GLSZM)	Default voxel array shift: 1,000,000
Neighboring Gray-Tone Difference Matrix (NGTDM)	Distance: [1]
Gray-Level Dependence Matrix (GLDM)	Distance: [1]; Angles: [0°, 45°, 90°, 135°]
2D shape descriptors (Shape2D)	Restricted to planar topological properties (for dimensional consistency)

1.2 Core Extraction Settings

- Histogram bin width: 25 (binWidth=25)
- Intensity normalization: Enabled (normalize=True)
- Analysis space: Enforced 2D (force2D=True)
- Texture feature connectivity: 8-pixel neighborhood (2D space)

- Angular relationships (for applicable textures): Calculated at 0°, 45°, 90°, 135°

1.3 Image Transformations for Derivative Features

Wavelet filtering (all decomposition levels), Laplacian of Gaussian (LoG) filtering, Square transformation, Square Root transformation, Logarithm transformation, Exponential transformation, Gradient magnitude.

2. Detailed Feature Dimensionality Reduction Steps

- Reliability pre-selection: Features with ICC ≤ 0.8 (inter- and intra-observer reliability) were excluded.
- Kruskal-Wallis test: Contrasted intergroup feature distribution; features with $p \geq 0.05$ were removed.
- Pearson correlation analysis: Calculated pairwise correlation coefficients; for feature pairs with $|r| > 0.9$, one feature was iteratively removed to avoid multicollinearity.
- LASSO regression: Shrunk feature coefficients to zero; only features with non-zero coefficients were retained as the final subset.

3. Hyperparameter Optimization for Machine Learning Models (Grid Search)

**Model**	**Optimized Hyper parameters**
Logistic Regression (LR)	Regularization strength (C: [0.1, 1, 10]); Penalty type (L2 or none)
Decision Tree (DT)	Tree depth (max_depth: [10, 15]); split constraints (min_samples_split: [2, 3]); leaf constraints (min_samples_leaf: [1, 2])

Random Forest (RF)	Ensemble size (n_estimators: [200, 300]); Tree depth (max_depth: [15, 20]); leaf constraints (min_samples_leaf: [1, 2])
Support Vector Machine (SVM)	Regularization (C: [1, 10, 100]); Kernel functions (linear/rbf); Gamma coefficients (scale/auto)
Gradient Boosting Machine (GBM)	Boosting iterations (n_estimators: [200, 300]); Learning rate (0.05/0.1); Tree depth (max_depth: [5, 7])
Extreme Gradient Boosting (XGB)	Boosting rounds (n_estimators: [100, 200]); Learning rate (0.05/0.1); Tree depth (max_depth: [3, 5]); multi-class parameterization (objective='multi:softprob', num_class dynamically set)

Table S1. Performance of machine learning models for liver fibrosis staging using liver 2D-SWE images in training and validation cohorts

		AUC	Sensitivity (%)	Specificity (%)	PPV (%)	NPV (%)	LR+	LR−
S4								
RF	T	1.00(1.00-1.00)	1.00	1.00	1.00	1.00	-	0.00
	V	0.98(0.95-1.00)	0.94	0.95	0.91	0.97	20.00	0.07
DT	T	0.99(0.99-1.00)	0.95	0.99	0.98	0.97	121.00	0.06
	V	0.89(0.75-1.00)	0.81	0.92	0.84	0.91	10.40	0.20
LR	T	0.97(0.96-0.99)	0.84	0.95	0.89	0.92	15.43	0.17
	V	0.89(0.82-0.96)	0.78	0.88	0.76	0.89	6.25	0.25
SVM	T	1.00(1.00-1.00)	1.00	1.00	1.00	1.00	-	0.00
	V	0.99(0.97-1.00)	0.88	0.97	0.93	0.94	28.00	0.13
GBM	T	1.00(1.00-1.00)	1.00	1.00	1.00	1.00	-	0.00
	V	0.98(0.94-1.00)	0.88	0.97	0.93	0.94	28.00	0.13
XGB	T	1.00(1.00-1.00)	1.00	1.00	1.00	1.00	-	0.00
	V	0.98(0.93-1.00)	0.81	0.92	0.84	0.91	10.40	0.20
⩾S3								
RF	T	1.00(1.00-1.00)	1.00	1.00	1.00	1.00	-	0.00
	V	0.99(0.98-1.00)	0.94	0.97	0.94	0.97	30.00	0.06
DT	T	0.99(0.99-1.00)	0.99	0.98	0.96	0.99	50.80	0.01
	V	0.92(0.81-1.00)	0.88	0.92	0.85	0.94	11.20	0.14
LR	T	0.97(0.95-0.99)	0.88	0.91	0.84	0.94	10.27	0.13

	V	0.92(0.83-1.00)	0.72	0.91	0.79	0.87	7.67	0.31
SVM	T	1.00(1.00-1.00)	1.00	1.00	1.00	1.00	-	0.00
	V	0.99(0.98-1.00)	0.94	0.97	0.94	0.97	30.00	0.06
GBM	T	1.00(1.00-1.00)	1.00	1.00	1.00	1.00	-	0.00
	V	0.99(0.99-1.00)	0.97	0.97	0.94	0.98	31.00	0.03
XGB	T	1.00(1.00-1.00)	1.00	1.00	1.00	1.00	-	0.00
	V	0.98(0.96-1.00)	0.88	0.94	0.88	0.94	14.00	0.13
⩾S2<br>RF	T	1.00(1.00-1.00)	1.00	1.00	1.00	1.00	-	0.00
	V	0.99(0.99-1.00)	0.97	1.00	1.00	0.98	-	0.03
DT	T	0.99(0.99-1.00)	0.98	0.99	0.98	0.99	84.00	0.02
	V	0.94(0.86-1.00)	0.91	0.95	0.91	0.95	19.33	0.10
LR	T	0.99(0.99-1.00)	0.98	0.99	0.98	0.99	125.00	0.02
	V	0.99(0.97-1.00)	1.00	0.97	0.94	1.00	32.00	0.00
SVM	T	1.00(1.00-1.00)	1.00	1.00	1.00	1.00	-	0.00
	V	1.00(1.00-1.00)	1.00	0.97	0.94	1.00	32.00	0.00
GBM	T	1.00(1.00-1.00)	1.00	1.00	1.00	1.00	-	0.00
	V	0.99(0.98-1.00)	0.97	0.97	0.94	0.98	31.00	0.03
XGB	T	1.00(1.00-1.00)	1.00	1.00	1.00	1.00	-	0.00
	V	0.99(0.98-1.00)	0.94	0.95	0.91	0.97	20.00	0.07

AUC, area under the receiver operating characteristic curve; LR+, positive diagnostic likelihood ratio; LR−, negative diagnostic likelihood ratio; NPV, negative predictive value; PPV, positive predictive value; T, training cohort; V, validation cohort; LR, logistic regression; DT, decision tree; RF, random forest; SVM, support vector machine; GBM, gradient boosting machine; XGB, extreme gradient boosting.

Table S2. Performance of machine learning models for liver fibrosis staging using spleen 2D-SWE images in training and validation cohorts

		AUC	Sensitivity (%)	Specificity (%)	PPV (%)	NPV (%)	LR+	LR−
S4								
RF	T	1.00(1.00-1.00)	1.00	1.00	1.00	1.00	-	0.00
	V	0.97(0.93-1.00)	0.83	0.93	0.87	0.91	12.78	0.18
DT	T	1.00(1.00-1.00)	1.00	1.00	1.00	1.00	-	0.00
	V	0.85(0.71-1.00)	0.75	0.96	0.90	0.88	17.25	0.26
LR	T	0.99(0.98-1.00)	0.92	0.98	0.96	0.96	42.96	0.08
	V	0.81(0.72-0.90)	0.67	0.98	0.94	0.85	30.67	0.34
SVM	T	1.00(1.00-1.00)	1.00	1.00	1.00	1.00	-	0.00
	V	0.98(0.93-1.00)	0.88	0.93	0.88	0.93	13.42	0.13
GBM	T	1.00(1.00-1.00)	1.00	1.00	1.00	1.00	-	0.00
	V	0.98(0.93-1.00)	0.83	0.98	0.95	0.92	38.33	0.17
XGB	T	1.00(1.00-1.00)	1.00	1.00	1.00	1.00	-	0.00
	V	0.98(0.93-1.00)	0.83	0.96	0.91	0.92	19.17	0.17
⩾S3								
RF	T	1.00(1.00-1.00)	1.00	1.00	1.00	1.00	-	0.00
	V	0.97(0.93-1.00)	0.83	0.96	0.90	0.92	19.41	0.18
DT	T	1.00(1.00-1.00)	1.00	1.00	1.00	1.00	-	0.00
	V	0.86(0.68-1.00)	0.83	0.89	0.79	0.91	7.77	0.19
LR	T	0.99(0.98-1.00)	0.97	0.98	0.96	0.98	44.76	0.03
	V	0.93(0.83-1.00)	0.91	0.89	0.81	0.95	8.58	0.10

SVM	T	1.00(1.00-1.00)	1.00	1.00	1.00	1.00	-	0.00
	V	0.99(0.97-1.00)	0.91	0.98	0.95	0.96	42.91	0.09
GBM	T	1.00(1.00-1.00)	1.00	1.00	1.00	1.00	-	0.00
	V	0.97(0.93-1.00)	0.78	0.96	0.90	0.90	18.39	0.23
XGB	T	1.00(1.00-1.00)	1.00	1.00	1.00	1.00	-	0.00
	V	0.98(0.95-1.00)	0.83	0.96	0.90	0.92	19.41	0.18
≥S2								
RF	T	1.00(1.00-1.00)	1.00	1.00	1.00	1.00	-	0.00
	V	0.99(0.98-1.00)	1.00	0.94	0.88	1.00	15.67	0.00
DT	T	1.00(1.00-1.00)	1.00	1.00	1.00	1.00	-	0.00
	V	0.90(0.72-1.00)	0.91	0.89	0.81	0.95	8.58	0.10
LR	T	0.99(0.99-1.00)	0.97	0.97	0.95	0.98	35.81	0.03
	V	0.97(0.89-1.00)	0.96	0.89	0.81	0.98	8.99	0.05
SVM	T	1.00(1.00-1.00)	1.00	1.00	1.00	1.00	-	0.00
	V	0.99(0.98-1.00)	0.96	0.96	0.92	0.98	22.48	0.05
GBM	T	1.00(1.00-1.00)	1.00	1.00	1.00	1.00	-	0.00
	V	0.99(0.98-1.00)	1.00	0.87	0.79	1.00	7.83	0.00
XGB	T	1.00(1.00-1.00)	1.00	1.00	1.00	1.00	-	0.00
	V	1.00(1.00-1.00)	1.00	0.91	0.85	1.00	11.75	0.00

AUC, area under the receiver operating characteristic curve; LR+, positive diagnostic likelihood ratio; LR−, negative diagnostic likelihood ratio; NPV, negative predictive

value; PPV, positive predictive value; T, training cohort; V, validation cohort; LR, logistic regression; DT, decision tree; RF, random forest; SVM, support vector machine; GBM, gradient boosting machine; XGB, extreme gradient boosting.

Table S3. Performance of machine learning models for liver fibrosis staging using liver grayscale images in training and validation cohorts

		AUC	Sensitivity (%)	Specificity (%)	PPV (%)	NPV (%)	LR+	LR−
S4								
RF	T	1.00(1.00-1.00)	1.00	1.00	1.00	1.00	-	0.00
	V	0.92(0.88-0.96)	0.83	0.86	0.75	0.90	5.95	0.20
DT	T	1.00(0.99-1.00)	1.00	0.99	0.99	1.00	461.00	0.00
	V	0.75(0.61-0.89)	0.74	0.77	0.61	0.85	3.16	0.34
LR	T	0.71(0.66-0.76)	0.55	0.75	0.52	0.77	2.16	0.61
	V	0.62(0.51-0.73)	0.52	0.71	0.48	0.75	1.80	0.68
SVM	T	0.99(0.99-1.00)	0.96	0.98	0.96	0.98	49.22	0.04
	V	0.86(0.79-0.93)	0.76	0.83	0.70	0.87	4.59	0.29
GBM	T	1.00(1.00-1.00)	1.00	1.00	1.00	1.00	-	0.00
	V	0.90(0.85-0.95)	0.81	0.88	0.77	0.90	6.66	0.22
XGB	T	1.00(1.00-1.00)	1.00	1.00	1.00	1.00	-	0.00
	V	0.89(0.84-0.95)	0.83	0.81	0.69	0.90	4.33	0.21
≥S3								
RF	T	1.00(1.00-1.00)	1.00	1.00	1.00	1.00	-	0.00
	V	0.88(0.83-0.93)	0.69	0.87	0.73	0.85	5.29	0.36
DT	T	1.00(1.00-1.00)	1.00	1.00	1.00	1.00	-	0.00
	V	0.68(0.53-0.80)	0.55	0.78	0.56	0.78	2.54	0.57
LR	T	0.68(0.63-0.73)	0.36	0.79	0.46	0.71	1.71	0.81

	V	0.62(0.52-0.73)	0.40	0.76	0.45	0.71	1.63	0.80
SVM	T	0.99(0.98-0.99)	0.95	0.98	0.97	0.97	62.42	0.05
	V	0.85(0.77-0.93)	0.62	0.85	0.68	0.82	4.20	0.45
GBM	T	1.00(1.00-1.00)	1.00	1.00	1.00	1.00	-	0.00
	V	0.87(0.81-0.93)	0.79	0.84	0.72	0.89	5.07	0.25
XGB	T	1.00(1.00-1.00)	1.00	1.00	1.00	1.00	-	0.00
	V	0.85(0.78-0.92)	0.72	0.83	0.69	0.86	4.38	0.33
≥S2 RF	T	1.00(1.00-1.00)	1.00	1.00	1.00	1.00	-	0.00
	V	0.81(0.71-0.91)	0.60	0.83	0.63	0.81	3.46	0.49
DT	T	1.00(0.99-1.00)	0.99	1.00	1.00	0.99	-	0.01
	V	0.56(0.43-0.70)	0.35	0.78	0.43	0.71	1.57	0.84
LR	T	0.63(0.58-0.68)	0.52	0.67	0.44	0.74	1.59	0.71
	V	0.55(0.44-0.67)	0.37	0.67	0.36	0.68	1.12	0.94
SVM	T	0.99(0.99-1.00)	0.96	0.97	0.94	0.98	31.58	0.04
	V	0.70(0.60-0.80)	0.47	0.74	0.47	0.74	1.83	0.71
GBM	T	1.00(1.00-1.00)	1.00	1.00	1.00	1.00	-	0.00
	V	0.83(0.74-0.92)	0.58	0.87	0.69	0.81	4.48	0.48
XGB	T	1.00(1.00-1.00)	1.00	1.00	1.00	1.00	-	0.00
	V	0.81(0.71-0.90)	0.47	0.87	0.64	0.77	3.66	0.60

AUC, area under the receiver operating characteristic curve; LR+, positive diagnostic likelihood ratio; LR−, negative diagnostic likelihood ratio; NPV, negative predictive value; PPV, positive predictive value; T, training cohort; V, validation cohort; LR, logistic regression; DT, decision tree; RF, random forest; SVM, support vector machine; GBM, gradient boosting machine; XGB, extreme gradient boosting.

Table S4. Performance of machine learning models for liver fibrosis staging using spleen grayscale images in training and validation cohorts

		AUC	Sensitivity (%)	Specificity (%)	PPV (%)	NPV (%)	LR+	LR−
S4								
RF	T	1.00(1.00-1.00)	1.00	1.00	1.00	1.00	-	0.00
	V	0.88(0.79-0.96)	0.74	0.86	0.72	0.87	5.20	0.30
DT	T	0.99(0.98-1.00)	0.99	0.96	0.92	0.99	23.00	0.01
	V	0.71(0.56-0.86)	0.63	0.79	0.59	0.81	2.93	0.47
LR	T	0.77(0.70-0.84)	0.59	0.77	0.56	0.79	2.56	0.54
	V	0.64(0.50-0.78)	0.49	0.66	0.41	0.72	1.42	0.78
SVM	T	0.99(0.99-1.00)	0.98	0.97	0.94	0.99	34.25	0.02
	V	0.78(0.68-0.88)	0.66	0.87	0.72	0.84	5.11	0.39
GBM	T	1.00(1.00-1.00)	1.00	1.00	1.00	1.00	-	0.00
	V	0.92(0.86-0.97)	0.80	0.90	0.80	0.90	8.00	0.22
XGB	T	1.00(1.00-1.00)	1.00	1.00	1.00	1.00	-	0.00
	V	0.89(0.82-0.96)	0.77	0.86	0.73	0.88	5.40	0.27
≥S3								
RF	T	1.00(1.00-1.00)	1.00	1.00	1.00	1.00	-	0.00
	V	0.84(0.75-0.92)	0.63	0.87	0.71	0.82	4.89	0.43
DT	T	0.99(0.98-0.99)	0.89	0.99	0.99	0.95	250.00	0.11
	V	0.73(0.58-0.88)	0.57	0.86	0.67	0.80	4.00	0.50
LR	T	0.65(0.59-0.72)	0.36	0.77	0.44	0.71	1.59	0.82

	V	0.60(0.46-0.74)	0.31	0.79	0.42	0.70	1.47	0.87
SVM	T	0.99(0.98-1.00)	0.94	0.99	0.97	0.97	66.00	0.06
	V	0.73(0.62-0.83)	0.60	0.90	0.75	0.82	6.00	0.44
GBM	T	1.00(1.00-1.00)	1.00	1.00	1.00	1.00	-	0.00
	V	0.87(0.80-0.95)	0.74	0.91	0.81	0.88	8.67	0.28
XGB	T	1.00(1.00-1.00)	1.00	1.00	1.00	1.00	-	0.00
	V	0.88(0.81-0.95)	0.66	0.96	0.88	0.85	15.33	0.36
≥S2 RF	T	1.00(1.00-1.00)	1.00	1.00	1.00	1.00	-	0.00
	V	0.80(0.70-0.90)	0.66	0.79	0.61	0.82	3.07	0.44
DT	T	0.99(0.99-1.00)	0.98	0.98	0.95	0.99	39.14	0.02
	V	0.58(0.42-0.74)	0.51	0.71	0.47	0.75	1.80	0.68
LR	T	0.76(0.70-0.82)	0.62	0.74	0.55	0.80	2.42	0.51
	V	0.70(0.58-0.83)	0.51	0.71	0.47	0.75	1.80	0.68
SVM	T	0.98(0.97-0.99)	0.94	0.98	0.95	0.97	37.71	0.06
	V	0.64(0.51-0.76)	0.66	0.69	0.51	0.80	2.09	0.50
GBM	T	1.00(1.00-1.00)	1.00	1.00	1.00	1.00	-	0.00
	V	0.82(0.70-0.94)	0.71	0.81	0.66	0.85	3.85	0.35
XGB	T	1.00(1.00-1.00)	1.00	1.00	1.00	1.00	-	0.00
	V	0.79(0.68-0.90)	0.71	0.76	0.60	0.84	2.94	0.38

AUC, area under the receiver operating characteristic curve; LR+, positive diagnostic likelihood ratio; LR−, negative diagnostic likelihood ratio; NPV, negative predictive value; PPV, positive predictive value; T, training cohort; V, validation cohort; LR, logistic regression; DT, decision tree; RF, random forest; SVM, support vector machine; GBM, gradient boosting machine; XGB, extreme gradient boosting.

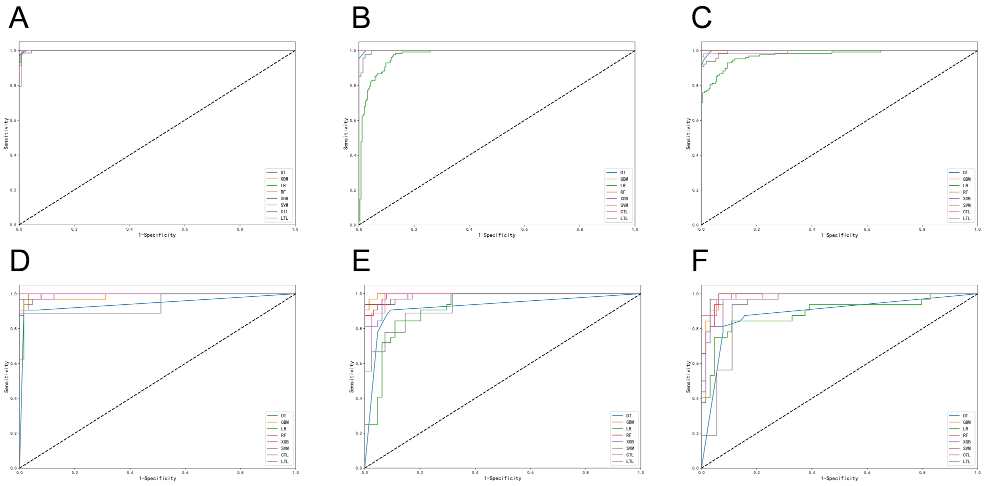

Figure S1. Comparison of ROC curves between ML and TL for liver fibrosis staging using liver 2D-SWE images in training and validation cohorts A,D: S0-S1 versus S2-S4 (≥S2) in training and validation cohorts; B,E: S0-S2 versus S3-S4 (≥S3) in training and validation cohorts; C,F: S0-S3 versus S4 (S4) in training and validation cohorts.

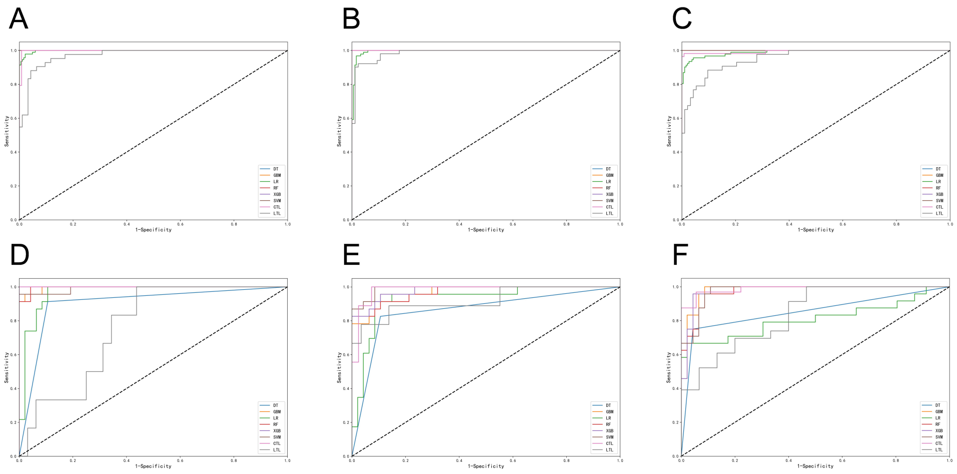

Figure S2. Comparison of ROC curves between ML and TL for liver fibrosis staging using spleen 2D-SWE images in training and validation cohorts A,D: S0-S1 versus S2-S4 (≥S2) in training and validation cohorts; B,E: S0-S2 versus S3-S4 (≥S3) in

training and validation cohorts; C,F: S0-S3 versus S4 (S4) in training and validation cohorts.

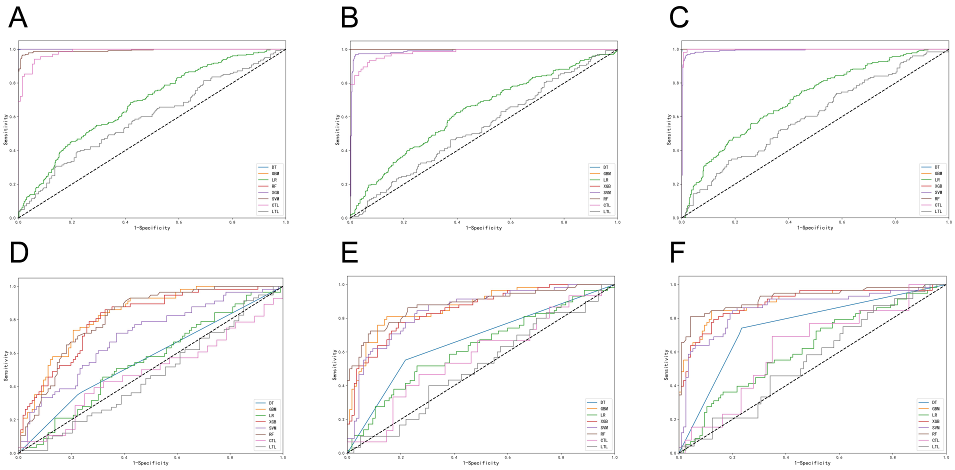

Figure S3. Comparison of ROC curves between ML and TL for liver fibrosis staging using liver grayscale images in training and validation cohorts A,D: S0-S1 versus S2-S4 (≥S2) in training and validation cohorts; B,E: S0-S2 versus S3-S4 (≥S3) in training and validation cohorts; C,F: S0-S3 versus S4 (S4) in training and validation cohorts.

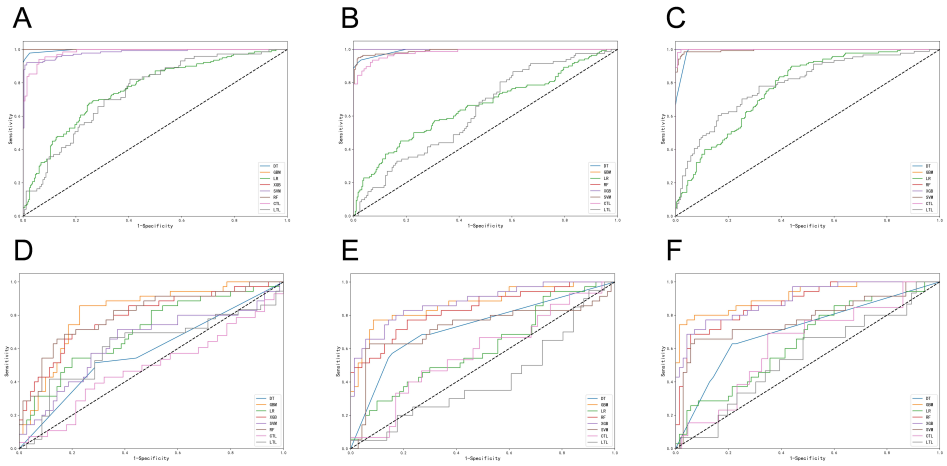

Figure S4. Comparison of ROC curves between ML and TL for liver fibrosis staging

using spleen grayscale images in training and validation cohorts A,D: S0-S1 versus S2-S4 (≥S2) in training and validation cohorts; B,E: S0-S2 versus S3-S4 (≥S3) in training and validation cohorts; C,F: S0-S3 versus S4 (S4) in training and validation cohorts.

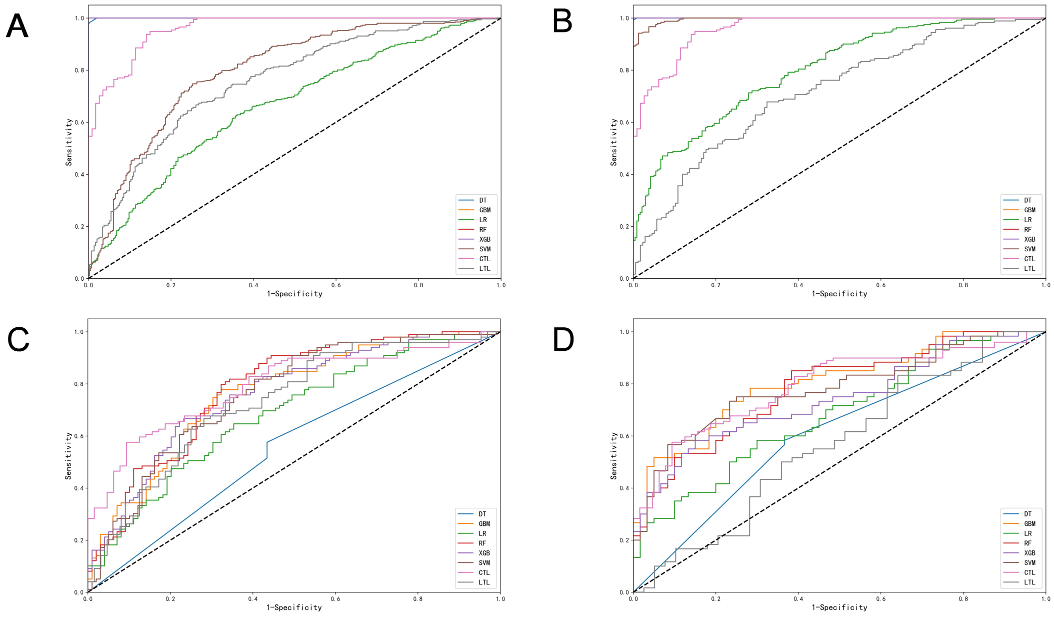

Figure S5. Comparison of ROC curves between ML and TL for etiology diagnosis using grayscale images in training and validation cohorts A,C: liver images in training and validation cohorts; B,D: spleen images in training and validation cohorts.
